# Direct-acting antivirals (DAA) positively affect depression and cognitive function in patients with chronic hepatitis C

**DOI:** 10.1371/journal.pone.0320221

**Published:** 2025-04-04

**Authors:** Tomasz Pawłowski, Marek Radkowski, Karol Perlejewski, Bogna Szymańska, Hanna Berak, Andrzej Horban, Tomasz Laskus

**Affiliations:** 1 Department of Psychiatry, Wrocław Medical University, Wrocław, Poland; 2 Department of Immunopathology of Infectious and Parasitic Diseases, Medical University of Warsaw, Warsaw, Poland; 3 Outpatient Clinic, Warsaw Hospital for Infectious Diseases, Warsaw, Poland; 4 Department of Adult Infectious Diseases, Medical University of Warsaw, Warsaw, Poland; Kaohsiung Medical University, TAIWAN

## Abstract

The aim of the study was to determine how depression and cognitive dysfunction in patients with chronic hepatitis C virus (HCV) infection are affected by treatment with direct-acting antivirals (DAA). Fifty-two chronic hepatitis C patients underwent neurocognitive and psychological evaluation before therapy and 5–6 months later. Depression was measured by Beck Depression Inventory (BDI), anxiety by State-Trait Anxiety inventory (STAI), neuroticism by Eysenck Personality Inventory (N/EPO-R), while Ruff Figural Fluency Test (RFFT), Wisconsin Card Sorting Test (WCST), The Grooved Pegboard Test (GPT), and California Verbal Learning Test (CVLT) were used to assess neurocognitive function. There was significant positive change in BDI scores (8.8 ± 6.6 vs 6.1 ± 6.1; p < 0.0001) while the most striking improvement in cognitive tests was observed for CVLT sum of immediate recall from Trial-1 to Trial-5 (50.9 ± 10.0 to 54.1 ± 10.0; p = 0.0005) and RFFT, where the number of unique designs increased from 77.2 ± 21.0 to 86.1 ± 28.3 (p < 0.0001). These differences remained significant when patients with advanced (METAVIR grade F3/F4) and those with mild (grade F0/F1/F2) liver disease were analyzed separately, although in general the improvements were more pronounced in the former group. In conclusion, in chronic HCV infection the brain function is markedly improved by DAA treatment.

## Introduction

The World Health Organization estimates that about 50 million individuals are chronically infected with hepatitis C virus (HCV) worldwide and about one million new infections occur per year [1], and other estimates put the number of infected at 56.8 million and the global prevalence of viraemic HCV infection at 0.7% [2]. HCV infection is a common etiologic factor of chronic hepatitis, liver cirrhosis, and hepatocellular carcinoma (HCC); [3]. However, beyond the risk of developing liver complications, patients with HCV infection have an increased risk of morbidity and mortality related to extrahepatic diseases including frequent and polymorphous autoimmune or lymphoproliferative disorders, cardiovascular (i.e., stroke, ischemic heart disease), renal, and metabolic complications but also those related to central nervous system [4].

Chronic HCV infection has been associated with depression and cognitive dysfunction [5–7], but the cause of these problems was believed for some time to be secondary to liver dysfunction and chronic illness. However, neurocognitive dysfunction in hepatitis C patients does not correlate completely with the severity of liver disease [6,8] and it was demonstrated by Forton and colleagues that hepatitis C patients have elevations of choline/creatine ratios in white matter and basal ganglia in proton magnetic-resonance spectroscopy (^1^H MRS) but such changes are not present in patients with hepatitis of other etiology or in healthy controls [6,9]. Since in hepatic encephalopathy the choline ratios are depressed [10], these changes were most likely directly related to HCV infection. Similar findings were later reported by others, which strengthens the argument that neurocognitive dysfunction and depression are not secondary to liver disease [8,11]. Importantly, very similar changes have been described in HIV-infected patients [12,13] and HIV is well-known to be neuroinvasive leading to dementia, minor neurocognitive disorder, and depression [14].

There is currently strong evidence that HCV is also neuroinvasive and neurotropic, although in contrast to HIV infection it does not cause dementia. First of all, negative-strand HCV RNA, which is viral replicative intermediate, was shown to be present in brain tissue collected at autopsy [15] and two subsequent studies detected HCV proteins in autopsy brain tissue using Western blotting and/or immunostaining [16,17]. In addition, brain-derived viral variants are often distinct from those circulating in blood and show tissue specific adaptations [15,18] thus likely to represent a separate compartment much like is the case in HIV infection. Furthermore, the access mode could also be similar - via infected leucocytes (‘Trojan horse’ phenomenon) and this assumption is supported by studies finding a close relationship between HCV sequences found in lymphoid cells and lymphoid tissues and those present in the CNS [15,18,19].

HCV may directly affect the central nervous system through release of proinflammatory cytokines by infected brain cells [20] and/or alterations in serotonergic and dopaminergic neurotransmission with resultant depressive symptoms, fatigue and cognitive impairment [21–23]. Furthermore, the neurocognitive changes could be due to the general inflammatory state directly related to chronic infection [24,25].

HCV eradication was found to improve some aspects of cognitive function and cerebral metabolism in patients treated with interferon and ribavirin [26,27] and recently similar findings were reported for patients undergoing therapy with direct-acting antivirals (DAA); [28]. Interestingly, the severity of depressive symptoms before treatment were recently reported to be predictive of successful treatment outcome [29].

The aim of the current prospective study was to determine the effect of DAA treatment in chronic hepatitis C patients on depression, anxiety, neuroticism, and a number of cognitive function tests.

## Methods

### Patients

The current analysis included patients who were being treated at the Outpatient Clinic of the Municipal Hospital for Infectious Diseases in Warsaw between January 2019 and February 2020. Eligible patients were ≥ 18 years old, have had detectable serum HCV-RNA and had neither history nor evidence of decompensated liver disease or liver encephalopathy. In addition, patients who were active drug or alcohol abusers or have been previously diagnosed with psychiatric illness (with the exception of depression as it could have been due to chronic HCV infection) were excluded. All recruited patients underwent psychological testing right before the initiation of antiviral therapy and repeated testing was conducted 5–6 months thereafter (3 months after the end of typical 12-week therapy).

While 80 patients were initially enrolled into the study, only 54 completed the second testing. However, two of these patients remained HCV-RNA positive and thus only 52 patients were included in the final analysis which required data from two time-points and positive treatment outcome. Both patients remaining HCV-RNA positive had cirrhosis, which is not unexpected since while DDA treatment success rate is often >  95% it is lower in patients with advanced disease [30]. Liver fibrosis was measured by FibroScan using five point METAVIR scale grading. In this scale F0/F1 represents no or minimal fibrosis, F2 moderate fibrosis, F3 severe fibrosis, and F4 represents cirrhosis [31]. Specific FibroScan cut-off values used were ≤  7 kPa for F0-F1, 7.1–9.4 kPa for F2, 9.5–12.4 kPa for F3, and ≥  12.5 kPa for F4.

Written informed consent was obtained from each study participant and the study protocol adhered to ethical guidelines of the Declaration of Helsinki. The study was approved by the Bioethical Committee of the Medical University of Wroclaw (KB-341/2006).

### Psychological evaluation

The patients’ mental state was assessed by psychological clinical examination and psychometric tests. All tests were administered by the same certified psychologist who was unaware of patients’ clinical data and treatment outcome. Depression was measured by the multiple choice Beck Depression Inventory (BDI); [32], neuroticism was evaluated by Eysenck Personality Inventory (N/EPO-R); [33] while anxiety was assessed by State-Trait Anxiety inventory (STAI); [34]. Cognitive function was measured with the help of The Ruff Figural Fluency Test (RFFT); [35], Wisconsin Card Sorting Test (WCST); [36], and California Verbal Learning Test (CVLT); [37]. For assessing motor functioning the Grooved Pegboard Test (GPT) was employed, separately for the dominant and non-dominant hand [38].

WCST measures cognitive flexibility, problem-solving skills, working memory, and abstraction. Since it is also sensitive to frontal lobe dysfunction it could be considered a measure of executive function. Responses are classified as correct responses, total errors, perseverative responses, perseverative errors, and non-perseverative errors. The RFFT was measured as a number of unique designs, while CVLT was recorded as the sum of immediate recall from Trial-1 to Trial-5. The former test assesses non-verbal fluency within the domain of executive functioning, and the latter evaluates verbal learning and memory.

### Statistical analysis

Continuous variables were summarized as means and standard deviation (SD) or median and interquartile range (IQR), and categorical as frequency and percentage. Nonparametric Wilcoxon matched-pairs signed rank test was employed to compare variables before and after treatment. P ≤ 0.05 was considered to be statistically significant. Calculations as well as graphs were done with the help of GraphPad Prism version 9.50 for Windows (GraphPad Software, San Diego, California, USA).

## Results

Out of the 80 initially enrolled patients who underwent neurocognitive testing before the initiation of treatment only 54 could be tested again after 6 months as 26 patients either refused future participation (20 patients) or did not show up for the arranged visit (6 patients). These 26 patients did not differ from the rest of the study group with regard to the baseline results of psychological and cognitive testing, treatment outcome, severity of liver disease, age, education level, and marital or employment status. The 20 patients who refused the second testing provided lack of time for such a lengthy testing procedure and/or travel difficulties as explanation.

The baseline characteristics of all 52 patients included in the final analysis are shown in Table 1. There were 29 women (56%) and 23 men (44%), their median age was 46.5 years (range 28–83 years) and the median time from the initial diagnosis of HCV infection was 4.0 years (range 1–26 years). Median pretreatment HCV viral load was 1.23 x 10^6^ IU/ml (interquartile range [IQR] 0.38–2.81 x 10^6^ IU/ml). Among 52 patients, 21 were classified as F0/F1, nine as F2, eight as F3 and 14 as F4 on the METAVIR scale. For the purpose of the current study patients with advanced disease (F3 and F4) were analyzed together. The most common HCV genotype was 1 as it was present in 44 (85%); two patients were infected with genotype 3 and 6 with genotype 4.

**Table 1:** Baseline characteristics of 52 patients with chronic hepatitis C undergoing treatment: ^a^median, interquartile range (IQR); ^b^Normal range 4–35 (U/l); SVR - sustained virological response.

Variables		All patients (n = 52)	Advanced LiverDisease (n = 22)	Mild Liver Disease (n = 30)
**Male (%)**		23 (44)	11 (50)	12 (40)
**Age (years)** ^**a**^		46.5 (38.5–63.0)	60.0 (43.0–67.0)	42.0 (37–56)
**Time since initial diagnosis in years; median (range)**		4 (1–26)	2 (1–26)	5 (1–23)
**Previous treatment (pegIFN+RBV)**		7	0	7
**Viral load (x10**^**6**^ **IU/mL)**^a^		1.23 (0.38–2.81)	1.54 (0.33–2.47)	1.07 (0.53–3.33)
**Genotype**	**1b/1a**	44	18	26
	**3**	2	1	1
	**4c/4d**	6	3	3
**Baseline ALT** ^a,b^		68 (48–102)	101 (79–146)	55 (42–76)
**SVR (%)**		52 (100)	22 (100)	30 (100)
**Treatment**				
**sofosbuvir** + **ledipasvir**		19	3	16
**sofosbuvir** + **ledipasvir** + **RBV**		4	4	0
**grazoprevir** + **elbasvir**		12	3	9
**glecaprevir** + **pibrentasvir**		2	2	0
**sofosbuvir** + **velpatasvir**		15	10	5

Patients were treated for 8 to 12 weeks with different combinations of direct-acting antivirals, which are listed in Table 1. All 52 patients achieved sustained virological response (SVR), which is defined as undetectable (or below the lower limit of quantification) HCV-RNA at 12 weeks after completion of treatment. Eight patients were previously unsuccessfully treated with interferon and ribavirin (3–5 years before the current treatment). Adverse events associated with treatment were fatigue in 13 (25%) patients, nausea in five (10%), and diarrhea in three (6%).

**Fig 1:**
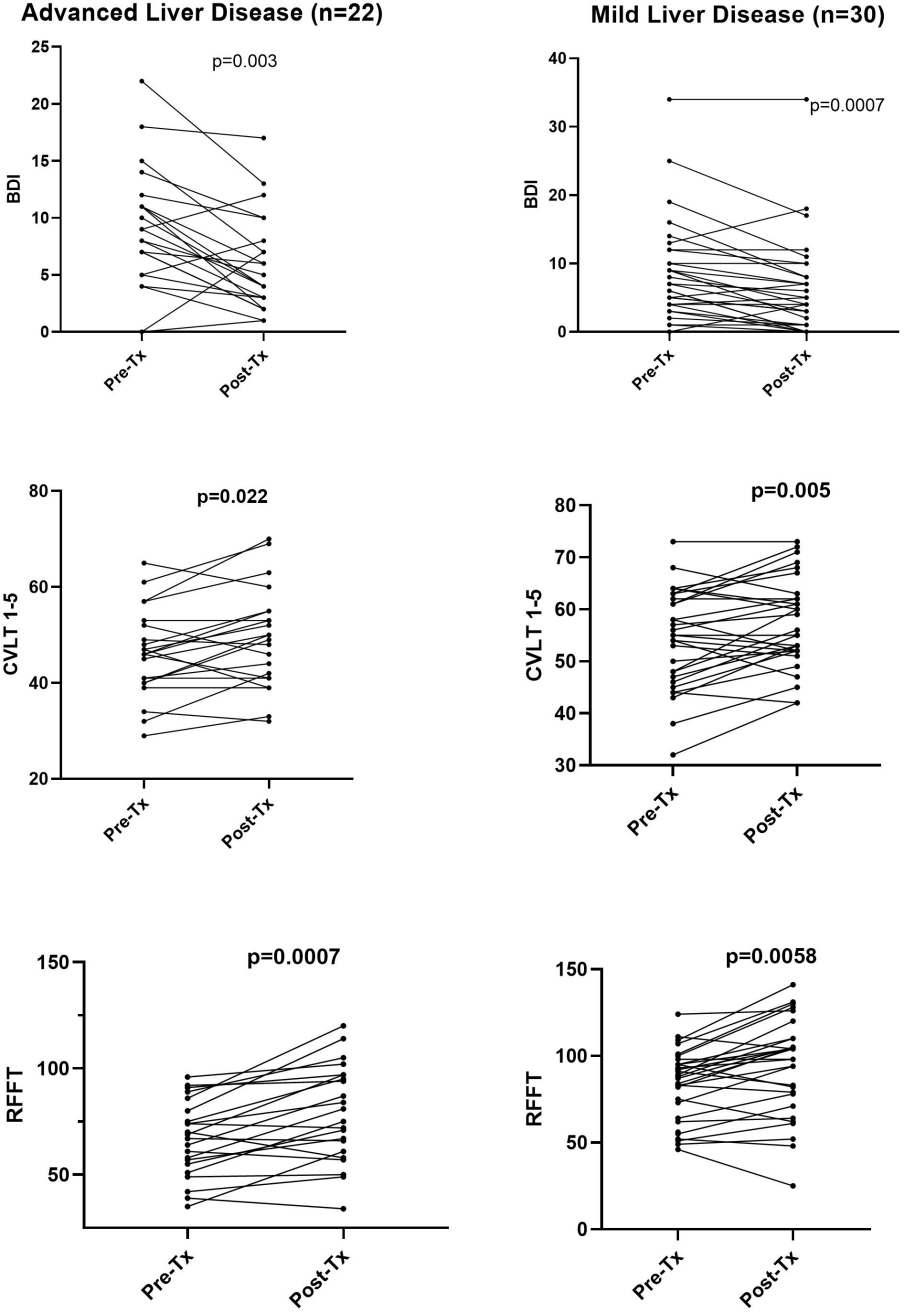
Depression and results of some cognitive tests in patients with chronic HCV infection before and after antiviral treatment. Patients with advanced liver disease (METAVIR grade F3/F4) and those with mild disease (METAVIR grade F0/F1/F2) are shown separately. Depression was measured by Beck Depression Index (BDI) and the cognitive tests presented are CVLT 1–5 and RFFT. Data were compared using the nonparametric Wilcoxon signed test.

Results of psychological and cognitive testing before and after therapy are shown in Table 2 and the most pronounced changes are also presented in Fig 1. Only those measurements that were considered most important were analyzed. Most strikingly, there was a highly significant drop in depression scores after the end of therapy (BDI 8.8 ± 6.6 vs 6.1 ± 6.1; < 0.0001). Although statistical significance was lower, similar lowering of BDI was present both in advanced liver disease group (8.9 ± 5.3 vs 5.9 ± 4.3) and mild liver disease patients (8.5 ± 7.4 vs 6.1 ± 7.1); (Table 2). However, as seen in Fig 1 in a minority of mild and advanced liver disease subjects the BDI scores increased or remained unchanged.

**Table 2:** Depression, anxiety, neuroticism and results of neurocognitive tests: ^a^Beck Depression Inventory; ^b^Assessed by State-Trait Anxiety inventory (STAI); ^c^Evaluated by Eysenck Personality Inventory (N/EPO-R); ^d^Sum of immediate recall from Trial-1 to Trial-5 in California Verbal Learning Test (CVLT); ^e^Ruff Figural Fluency Test (RFFT); ^f^Wisconsin Card Sorting Test (WCST) in 52 chronic hepatitis C patients before and after antiviral therapy. Patients with advanced liver disease were F3/F4 on the Metavir scale and patients with mild liver disease were F0-F3. Statistical analysis was conducted using Wilcoxon matched-pairs signed rank test.

Parameter	All Patients (n = 52)			Advanced Liver Disease (n = 22)			Mild Liver Disease (n = 30)		
	Before treatment	After treatment	P value	Before treatment	After treatment	P value	Before treatment	After treatment	P value
**BDI** ^a^	8.8 ± 6.6	6.1 ± 6.1	<0.0001	8.9 ± 5.3	5.9 ± 4.3	0.003	8.5 ± 7.4	6.1 ± 7.1	0.0007
**Anxiety** ^b^	33.6 ± 8.2	32.7 ± 8.9	0.19	34.5 ± 6.1	31.0 ± 7.5	0.032	33.0 ± 9.3	33.6 ± 9.6	0.94
**Neuroticism** ^c^	5.5 ± 5.0	4.5 ± 3.2	0.045	3.9 ± 3.0	3.7 ± 2.8	0.54	6.5 ± 5.6	5.0 ± 3.2	0.06
**CVLT 1-5** ^d^	50.9 ± 10.0	54.1 ± 10.0	0.0005	46.1 ± 9.1	49.3 ± 10.3	0.022	53.6 ± 10.0	57.2 ± 8.6	0.005
**RFFT**^e^ **(unique designs)**	77.2 ± 21.0	86.1 ± 28.3	<0.0001	67.0 ± 17.8	78.9 ± 22.7	0.0007	83.6 ± 20.6	90.9 ± 30.8	0.006
**WCST** ^f^ **(total correct answers)**	76.3 ± 12.7	75.0 ± 10.1	0.09	77.8 ± 14.8	78.1 ± 9.1	0.33	75.4 ± 10.9	72.5 ± 10.2	0.14
**WCST**^f^ **(total errors)**	28.9 ± 14.2	24.5 ± 14.7	0.025	32.0 ± 12.5	26.3 ± 11.7	0.016	26.7 ± 15.0	23.0 ± 16.4	0.28
**WCST**^f^ **(perseverative errors)**	15.6 ± 8.8	12.6 ± 8.7	0.005	17.9 ± 7.3	14.4 ± 7.1	0.031	14.0 ± 9.3	11.2 ± 9.5	0.064
**Grooved pegboard test (dominant hand)**	70.4 ± 24.6	69.0 ± 18.3	0.63	78.6 ± 34.5	74.8 ± 16.1	0.93	64.5 ± 10.4	64.6 ± 18.6	0.25
**Grooved pegboard test** **(non-dominant hand)**	79.4 ± 21.0	77.3 ± 25.2	0.31	87.6 ± 26.7	86.6 ± 24.9	0.92	73.7 ± 13.1	70.7 ± 23.1	0.088

When pre- and post-treatment scores were compared, the anxiety was also lowered but these differences were less pronounced and reached statistical significance only in the group of patients with advanced liver disease (34.5 ± 6.1 vs 31.0 ± 7.5; p = 0.032). Interestingly, while there was no improvement in neuroticism among advanced liver disease patients, there was some positive change in those with milder forms of liver disease (6.5 ± 5.6 vs 5.0 ± 3.3) but this difference did not reach statistical significance (p = 0.06).

Among cognitive tests the most striking improvement was observed for CVLT 1-5, which is the sum of immediate recall from Trial-1 to Trial-5 and which assesses verbal learning and memory. This score increased from 50.9 ± 10.0 to 54.1 ± 10.0 (p = 0.0005) for all patients and the change was also significant in advanced liver disease patients (46.1 ± 9.1 vs 49.3 ± 10.3; p = 0.022) and mild disease subjects (53.6 ± 10.0 vs 57.2 ± 8.6; p = 0.005); (Table 2 and Fig 1).

Similar improvement was also observed in the RFFT score, which measures non-verbal fluency domain within executive functioning domain. The number of unique designs increased from 77.2 ± 21.0 to 86.1 ± 28.3 (p < 0.0001) and the difference was more pronounced in advanced liver disease (67.0 ± 17.8 vs 78.9 ± 22.7; p = 0.0007) than in mild liver disease patients (83.6 ± 20.6 vs 90.9 ± 30.8; p = 0.0058).

When WCST responses were analyzed, treatment improved the number of total and perseverative errors and these differences seemed to be slightly more pronounced in patients with more advanced liver disease (Table 2). Finally, treatment did not seem to have any significant effect on the motor functioning as assessed separately for the dominant and non-dominant hand by the GPT (Table 2).

## Discussion

In our prospective study of 52 chronic hepatitis C patients, DAA treatment resulted in significant improvement in depression scores and results of several neurocognitive tests and these differences were more pronounced in patients with more advanced liver disease (METAVIR grade F3/F4). Since patients in the latter group were significantly older, it seems that the positive effect of treatment is not confined to younger patients. However, it is unclear whether this effect was due directly to viral eradication or was secondary to suppression of inflammation.

There is strong evidence that HCV is neurotropic as postmortem studies demonstrated the presence of viral proteins and viral replicative forms in brain tissue [15–17]. Two independent groups identified infected cell as being astrocytes and macrophages/microglia [16,17] and it was later shown that the latter cells are activated and express a number of proinflammatory cytokines [20]. However, the neurotransmission and neuroplasticity in mood regulating brain regions seems to be affected by any chronic inflammatory process [24,39]. The proinflammatory cytokines were found to activate the tryptophan–kynurenine pathway resulting in the production of the neurotoxic N-methyl-D-aspartate (NMDA) glutamate agonist quinolinic acid and 3-hydroxykynurenine [39] and they can also affect the re-uptake of monoamine neurotransmitters by neuronal mitogen-activated protein kinase (MAPK) regulation. The latter leads to increased surface expression of monoamine transporters on neurons [40]. Furthermore, such drugs as celecoxib, which is cyclooxygenase 2 inhibitor, and tetracycline antibiotic minocycline, which is an inhibitor of microglial inflammatory activation, were found to have antidepressant effects [41,42]. There is also experimental evidence that standard selective serotonin re-uptake inhibitors (SSRI) have another potential mode of action since they reduce the release of cytokines from activated macrophages and microglia [43].

While neurocognitive impairment in chronic HCV infection has been demonstrated in a number of studies [44], it is still unclear whether it is related to virus, liver injury, inflammatory state, or even to minimal hepatic encephalopathy (MHE). In support of the latter are findings by Hilsabeck and colleagues who found that cognitive performance was negatively associated with the stage of fibrosis [45]. MHE was likely present among our patients since those with more advanced liver disease had generally worse performance scores than patients with milder forms both before and after treatment (Table 2). To remedy the confounding effect of MHE on cognitive impairment, anxiety and depression, Weissenborn *et al.* [11] conducted parallel MRS testing which excluded MHE presence. The role of systemic inflammation in neurocognitive impairment is supported by the study of Tan et al. [46] who found erythrocyte sedimentation rate (ESR), to be a significant mediator of attention deficits in HCV patients. The same study compared patients with chronic hepatitis C to patients with chronic hepatitis B. While individuals with chronic hepatitis C showed deficits in executive functions, psychomotor speed, memory, and attention, patients with chronic hepatitis B showed deficits in language and executive function. These results suggest diverging pathogenic mechanisms of chronic HBV and HCV on neurocognitive functions.

The likely important role of inflammation in cognitive impairment among HCV-infected patients is further supported by a recent study demonstrating that the effect of TGFB1 polymorphism on cognitive functions is influenced by the presence of viral hepatitis [47]. Specifically, in healthy controls, the rs2241715 polymorphism was found to affect MMSE and MoCA language functions, while the rs10417924 polymorphism affected the “orientation to time” task in the MMSE. These effects were negatively modified by the presence of viral hepatitis which suggests that genetic susceptibility could be a key factor influencing cognitive deficits encountered in chronic HCV infection and could explain why the response to viral eradication is so inconsistent.

However, it should be mentioned that some studies have found no impairment in cognitive function in HCV-infected patients. In the study by Hilsabeck *et al.* [45] test scores of patients with chronic hepatitis C were similar to those of patients with other chronic liver diseases, but the control group was small. In still another study patients with compensated cirrhosis or chronic hepatitis, despite unimpaired neuropsychological tests, demonstrated a decrease in the quality of life [18].

The positive effects of treatment-induced viral eradication were first reported for pegylated interferon and ribavirin. In a small prospective study, which included both HIV/HCV coinfected and HCV monoinfected patients, treatment success was associated with improvement in some, but not all, measures of cognitive function [26]. In another small study successful treatment was followed by reductions in basal ganglia Cho/Cr and basal ganglia MI/Cr, which is compatible with reduced brain infection and/or immune activation. Patients who cleared the virus showed also improvements in memory, verbal learning, and visuospatial memory [27]. In our previous study, which was limited to analysis of depression and neuroticism, there were no significant changes after the end of interferon/ribavirin therapy [48]. Similarly, in their cross-sectional study including both interferon/ribavirin and DAA treated patients Tan et al. [46] found that successful elimination of hepatitis C resulted in improved liver function, but not neuropsychological test performance. However, the administration of interferon *per se* could activate inflammatory responses in the brain [49–52] and thus could have affected the results of the latter studies.

Since the introduction of DAA therapy, several studies reported on its effects on the results of neuropsychological testing. However, the very high success rate of this therapy made it practically impossible to compare between patients who achieve and those who don’t achieve SVR. In the first such study there was a significant improvement in neurophysiological (critical flicker frequency; CFF) but not in neurocognitive (number connection and digital symbol) tests, but the study was limited to 25 patients [53]. Two larger studies, which were published recently, provided more conclusive answers to the effects of DAA therapy on neurocognitive function.

In the first of these reports patients who achieved SVR had significant reduction in BDI, and anxiety, and showed improvement in computer-administered tests for visual memory, number connection, Stroop test and reaction times. However, since the study was conducted in India the dominant genotype was 3 and few women were included [54]. The second study, which enrolled Spanish patients, found improvement in CFF, motor and executive function, working memory and global cognitive function irrespective whether cirrhosis was present or not [28]. Interestingly, it was reported that DAA treatment might result in white matter tracts recovery, most likely by reducing neuroinflammation [55,56]. However, it should be emphasized that, similarly to the results of our study, the treatment-related improvements did not affect all analyzed domains.

Two recent studies provided evidence of beneficial effects of DAA treatment on depression. Abdel Moez *et al.* [57] analyzed 150 patients using BDI and showed sequential improvement in scores at one and three months post therapy when compared to pretreatment baseline. Interestingly, elderly females with advanced liver fibrosis were least likely to benefit from treatment. Progressive decline in the Hamilton Depression rating scale (HDS) scores during and three months after the end of therapy was also reported by Mahran *et al.* [58] who studied 170 patients undergoing DAA therapy; in this study anxiety scores improved in parallel regardless of gender. However, in striking contrast to the above reports and our own observations, two other studies found DAA effect to be detrimental with respect to depression. Egmond *et al.* [59] analyzed 54 patients who were euthymic at baseline and found that during treatment 13% and 46% developed major depression and any depressive disorder, respectively. However, 80% of patients received ribavirin, which could have some negative mental effects, and there was no follow up after treatment as the last time-point of analysis was the end of therapy. Khalil *et al.* [60] analyzed 47 patients and found that BDI scores increased significantly at 12 weeks after DAA therapy when compared to pretreatment values and 32% of subjects developed moderate to severe depression. However, most of these patients were illiterate or received only low education and their socioeconomic status was very low. Thus, the depression could have been related to the stigma of hepatitis diagnosis and financial burden of the disease as many participants had difficulties in work adjustment or even lost their jobs during the time of treatment.

Our study has three obvious shortcomings. First, the number of patients was limited and they were not homogenous with respect to the grade of liver fibrosis. Second, we could not totally exclude the effects of learning as the same tests were repeated. However, the tests were administered only twice and 5–6 months apart which lowers such a possibility. Third, while 80 patients were initially enrolled into the study, only 54 completed the second testing.

In conclusion, the depression and cognitive function in chronic HCV infection are improved by DAA treatment, particularly among patients with advanced liver disease. However, it remains unclear whether this is due to direct suppression of viral replication or is secondary to the overall lowered inflammation.
